# Uncovering new insights into the role of the ubiquitin ligase Smurf1 on the regulation of innate immune signaling and resistance to infection

**DOI:** 10.3389/fimmu.2023.1185741

**Published:** 2023-05-09

**Authors:** Luiz Pedro Souza-Costa, Josiane Teixeira Andrade-Chaves, Juvana Moreira Andrade, Vivian Vasconcelos Costa, Luis Henrique Franco

**Affiliations:** ^1^ Departamento de Bioquímica e Imunologia, Instituto de Ciências Biológicas, Universidade Federal de Minas Gerais, Belo Horizonte, Brazil; ^2^ Departamento de Morfologia, Instituto de Ciências Biológicas, Universidade Federal de Minas Gerais, Belo Horizonte, Brazil

**Keywords:** ubiquitin ligase, ubiquitination, Smurf1, innate immunity, infection

## Abstract

Innate immunity is the body’s first line of defense against infections. Innate immune cells express pattern recognition receptors in distinct cellular compartments that are responsible to detect either pathogens-associated molecules or cellular components derived from damaged cells, to trigger intracellular signaling pathways that lead to the activation of inflammatory responses. Inflammation is essential to coordinate immune cell recruitment, pathogen elimination and to keep normal tissue homeostasis. However, uncontrolled, misplaced or aberrant inflammatory responses could lead to tissue damage and drive chronic inflammatory diseases and autoimmunity. In this context, molecular mechanisms that tightly regulate the expression of molecules required for the signaling of innate immune receptors are crucial to prevent pathological immune responses. In this review, we discuss the ubiquitination process and its importance in the regulation of innate immune signaling and inflammation. Then, we summarize the roles of Smurf1, a protein that works on ubiquitination, on the regulation of innate immune signaling and antimicrobial mechanisms, emphasizing its substrates and highlighting its potential as a therapeutic target for infectious and inflammatory conditions.

## 1 Introduction

The innate immune system is the first line of defense against microbial infections. In addition to chemical and physical components, innate immunity is composed of cells such as macrophages, dendritic cells, and neutrophils, which sense the presence of infectious pathogens and activate inflammatory responses required for their elimination. The recognition of molecular components from pathogens by pattern recognition receptors (PRR) triggers a variety of intracellular signaling cascades in innate immune cells with subsequent activation of transcription factors that migrate to the nucleus and command the transcription of genes related to inflammatory and antimicrobial responses (1). Intracellular signaling triggered by innate immune receptors is a dynamic process composed of a complex combination of regulatory mechanisms that tightly regulate protein-protein interaction, protein subcellular localization, and protein abundance. One such regulatory mechanism is ubiquitination (2), a reversible post-translational modification that consists of the conjugation of ubiquitin to lysine residues in substrates (3). Substrates bound to ubiquitin are targeted to the proteasome for degradation or may interact with other proteins to play key physiological processes (4). In innate immune signaling, ubiquitination regulates the fate of substrates that actively work on signal transduction of PRR, and fine-tunes inflammatory immune responses to avoid tissue damage (5). In this review, we summarize the role of Smurf1, a protein required for the process of ubiquitination, on the regulation of innate immune signaling and antimicrobial mechanisms, emphasizing its substrates and highlighting its potential as a therapeutic target for infectious and inflammatory diseases.

## 2 Ubiquitination and ubiquitin ligases

**Figure 1:**
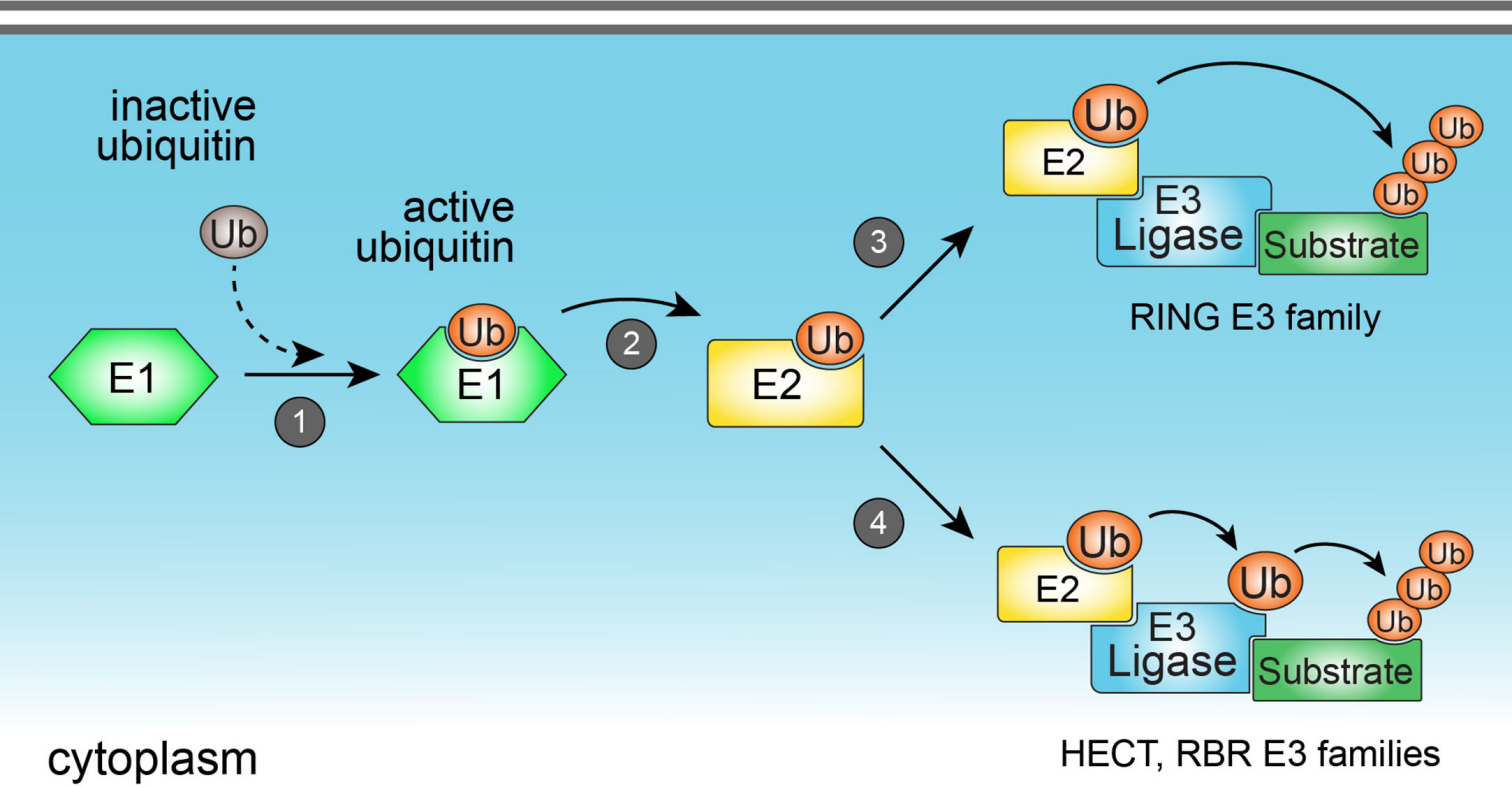
The ubiquitination process. Ubiquitination is catalyzed by the sequential action of three classes of enzymes (E1, E2, and E3 enzymes). Ubiquitination starts with the activation of cytoplasmic ubiquitin by the E1 ubiquitin-activating enzyme (1), followed by the transfer of activated ubiquitin to the E2 ubiquitin-conjugating enzyme (2). E2 enzyme may form a complex with the E3 ubiquitin ligase to promote the direct (3) (in the case of members from RING E3 ligase family), or indirect (4) (in the case of HECT and RBR E3 ligase families) transfer of ubiquitin to a specific lysine residue in a protein substrate.

Ubiquitination is a reversible enzymatic modification consisting of the covalent attachment of ubiquitin to target substrates, including proteins (3) and lipids (6). The ubiquitination process is crucial to regulate a number of cellular functions such as protein homeostasis, gene transcription, DNA repair and replication, intracellular traffic, and autophagy (7, 8). In the immune system, ubiquitination precisely regulates immune functions and signaling of a diverse set of cells including B and T lymphocytes, and innate immune cells (9). Ubiquitination is catalyzed by the sequential and orchestrated action of three classes of enzymes known as E1, E2, and E3 enzymes. The first step of ubiquitination consists of the activation of cytoplasmic ubiquitin by the E1 ubiquitin-activating enzyme, followed by the transfer of activated ubiquitin to the E2 ubiquitin-conjugating enzyme. Lastly, E2 conjugating enzyme may form a complex with the E3 ubiquitin ligase to promote the direct or indirect transfer of ubiquitin to a specific lysine residue in a protein substrate (**Figure 1**). During the last step of ubiquitination, E3 ubiquitin ligases interact directly with substrates and play a critical role in determining the specificity of ubiquitin attachment to them (10). Therefore, the enzymatic activity of E3 ubiquitin ligases is a key factor commanding the specificity of substrate ubiquitination. As the human genome codes around 600 E3 ubiquitin ligases (11), and considering the vast repertoire of intracellular substrates that are targeted for ubiquitination, it is reasonable to assume that each E3 ubiquitin ligase is capable to interact with and ubiquitinate more than one substrate, implicating them as key mediators of cellular homeostasis. For this reason, host E3 ubiquitin ligases have become attractive targets for pharmaceutical development of drugs against infectious, inflammatory, and tumor diseases (12–14).

**Figure 2:**
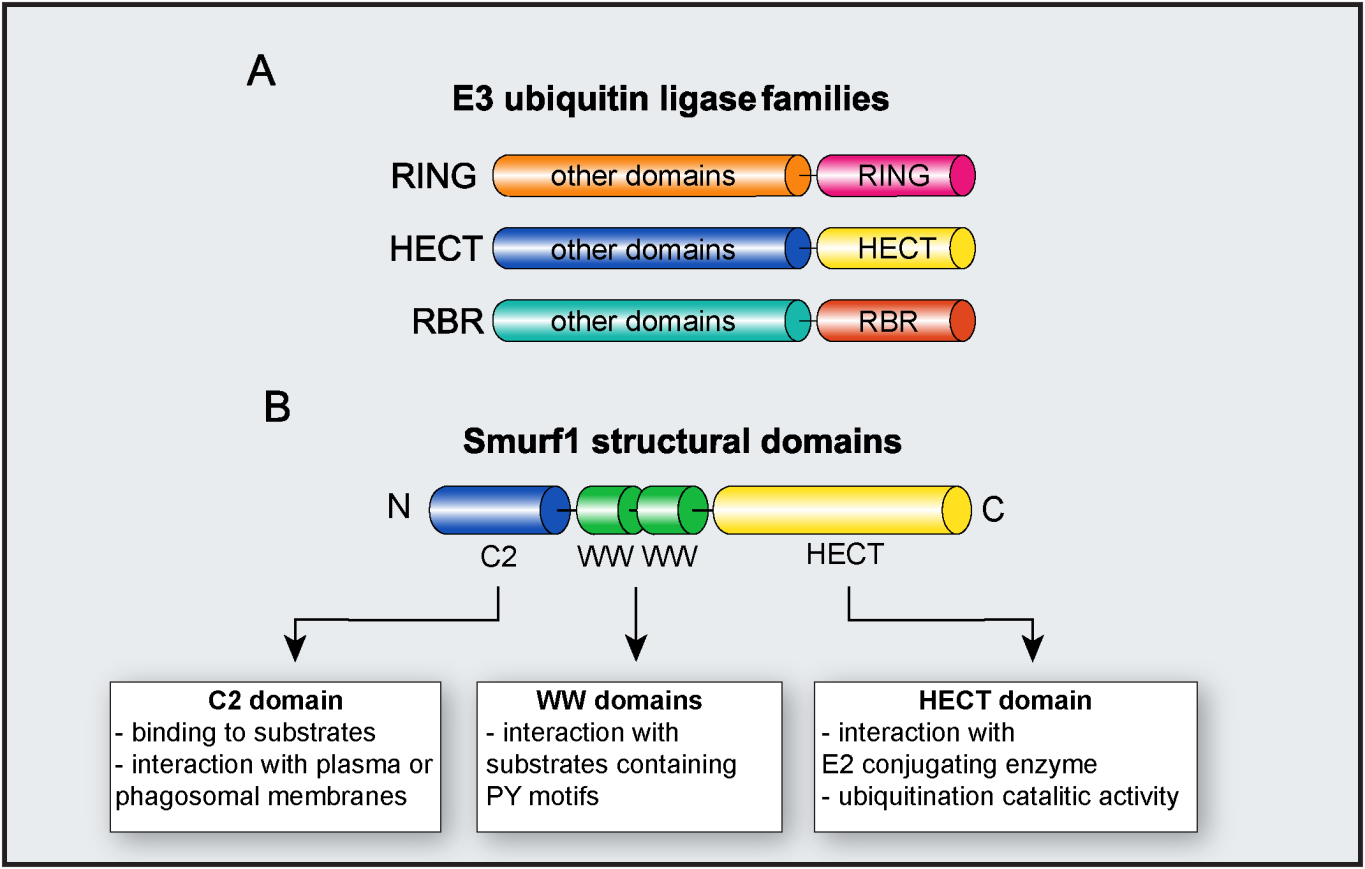
E3 ubiquitin ligase Smurf1. **(A)** Major families of E3 ubiquitin ligases. The diagram depicts RING, HECT, and RBR families and their respective functional domains. “Other domains” indicate variable domains, specific to each family of E3 ligase. **(B)** Molecular structure of Smurf1 and its identified domains.

E3 ubiquitin ligases are grouped into three families, according to their structure and mechanisms of action: Really interesting new gene (RING), homologous to the E6AP carboxy terminus (HECT), and ring-in-between-ring (RBR) (**Figure 2**). While E3 ubiquitin ligases of the RING family catalyze the direct transfer of ubiquitin from the E2 conjugating enzyme to the substrate (15), E3 ligases of either HECT and RBR families feature an intermediate step in which ubiquitin is first transferred from E2 to E3 ligase before being attached to the substrate (16, 17). E3 ligases from the HECT family are further classified into three subfamilies: Neuronal precursor cell-expressed developmentally downregulated 4 (NEDD4); HECT and RLD domain containing E3 ubiquitin protein ligase 2 (HERC); and other members of the HECT family (18). From these three subfamilies, members from NEDD4 are the most well-studied and characterized, and all of them share an N-terminal C2 domain (required for their binding to either phospholipids or substrates), two to four central WW domains (for binding to substrates), and a C-terminal HECT domain, responsible for their enzymatic activity (19). HERC and other members of the HECT family share the same HECT domain, with distinct substrate binding domains (18).

Following the ligation of the first ubiquitin residue on the substrate mediated by the E3 ubiquitin ligase, additional ubiquitin chains may be sequentially added into the lysine residues of the initial ubiquitin, resulting in the formation of polyubiquitin chains. Ubiquitin protein is composed of seven lysine residues (K6, K11, K27, K29, K33, K48, and K63) that are responsible for forming distinct topologies of polyubiquitin chains. The nature of each polyubiquitin chain regulates several homeostatic processes, and it dictates the substrate fate in the cell (20). K48-linked polyubiquitinated substrates are generally directed for proteasomal degradation and it represents the most abundant polyubiquitin topology found in the cells. The second most abundant and characterized polyubiquitin topology is the K63-linked polyubiquitin (4). The coupling of K63-linked polyubiquitin to substrates leads to the formation of a docking site that allows for the recruitment of downstream interactors that work in subsequent intracellular signaling pathways (21). The functions of the other polyubiquitin linkages and their roles in the regulation of cellular processes are currently under investigation (22).

## 3 E3 ubiquitin ligase Smurf1

SMAD ubiquitin regulatory factor 1 (Smurf1) is an E3 ubiquitin ligase belonging to the NEDD4 subfamily of E3 ligases (18). Smurf1 was first identified in 1999 as a factor capable to target for ubiquitination and proteasomal degradation of SMAD family member 1 (Smad1) and Smad5 during cellular responses to *bone morphogenetic protein* (BMP) (23). In subsequent years, several groups have identified Smurf1 substrates required for the regulation of a plethora of physiological functions including bone formation, osteoblast differentiation, cell growth and migration, cell adhesion and polarity, embryonic development (24), and selective autophagy (25, 26). Smurf1 has also been implicated in tumor development by ubiquitination of cancer-suppressing proteins (27, 28), in cancer metastasis by suppression of epithelial-mesenchymal cell transition (EMT) pathway (29–31), in pancreatic cancer invasiveness (32, 33), cardiovascular diseases (34), and in liver steatosis (35).

As a member of the NEDD4 subfamily, Smurf1 is composed of an N-terminal C2 domain, two central WW domains, and a C-terminal HECT domain (18) (**Figure 2**). The Smurf1 C2 domain contains a phospholipid-binding sequence that mediates Smurf1 interaction with plasma or phagosomal membranes (26, 36). Smurf1 C2 domain may also facilitate its interaction with substrates such as Ras homolog family member A (RhoA) and Axin (36, 37). WW domains are required for Smurf1 interaction with substrates containing PY motifs, and phosphorylation of substrates may increase their affinity of ligation with Smurf1 (38). Smurf1 HECT domain interacts with both E2 conjugating enzyme and ubiquitin during ubiquitination, and it is required for Smurf1 catalytic activity. The importance of the HECT domain in Smurf1 ubiquitin ligase activity is evidenced by findings that the C699A point mutation in the HECT domain completely abrogates its enzymatic activity (39, 40).

**Figure 3:**
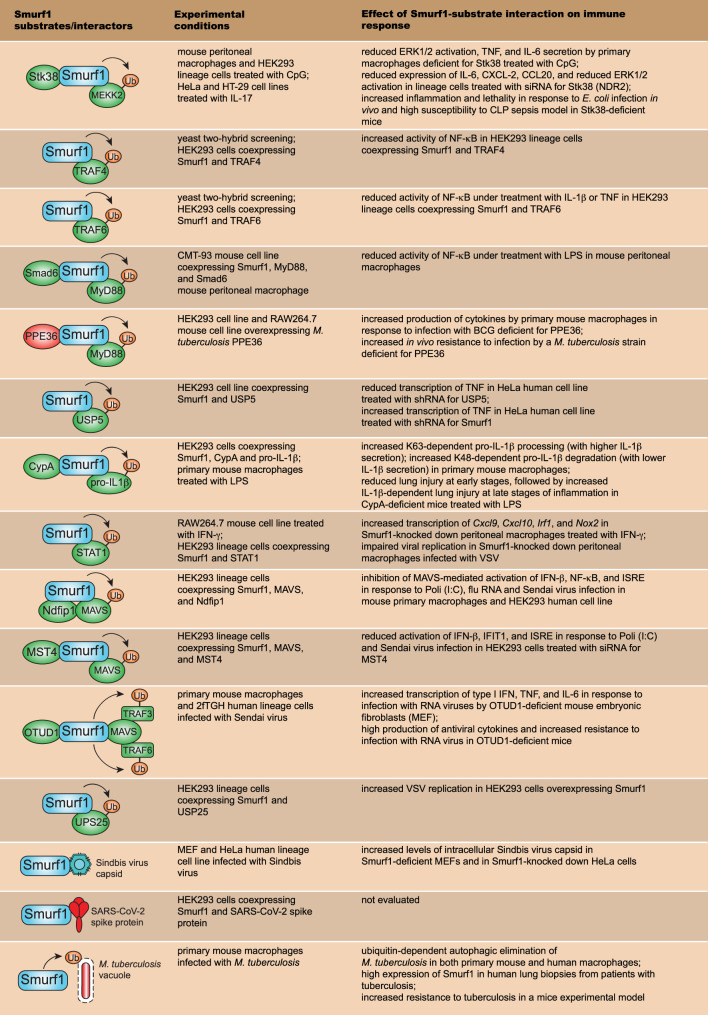
Smurf1 substrates that work on innate immune signaling pathways and resistance to infection. The first row depicts major Smurf1 substrates and interactors identified thus far; the second row details the specific experimental conditions in which Smurf1 interaction with substrates was identified; the third row shows the effect of Smurf1 interaction with a specific substrate on immune responses and susceptibility to infections.

Besides being involved in physiological and pathological processes, Smurf1 has been implicated in the regulation of innate immune signaling and control of microbial replication. In the next sections, we will summarize major substrates and processes related to immune responses and host resistance to infection that are directly regulated by Smurf1. **Figure 3** shows all Smurf1 substrates and interactors discussed in this review, highlighting the experimental conditions in which they were described, as well as the effect of their interaction with Smurf1 on the regulation of innate immune response and resistance to infections.

## 4 Regulation of innate immune signaling and inflammation by Smurf1

Innate immunity is characterized by a rapid response to infection and contributes to the induction of adaptive immune responses. PRRs are key components of innate immunity that have a main function to detect and initiate immune responses against microbial invasion. Innate immune cells including macrophages, neutrophils, and dendritic cells, express PRRs in distinct cellular compartments that detect not only molecular patterns associated with pathogens (PAMPs) but also molecules secreted by damaged or dead cells (DAMPs) (41). The engagement of PRR by PAMP or DAMP triggers signaling intracellular cascades that result in the activation of transcription factors that migrate to the nucleus and promote the transcription of genes related to inflammatory and antimicrobial responses (42). Toll-like receptors (TLR) are the most well-studied and characterized family of PRRs (41). TLR family is composed of 10 members in humans (TLR1-TLR10) and 12 members in mice (TLR1-TLR12). This family recognizes lipids, proteins, lipoproteins, and nucleic acids derived from a broad range of microbes such as bacteria, viruses, parasites, and fungi (43). The engagement of TLRs by microbial-derived components triggers the recruitment of adapter molecules to initiate downstream signaling pathways. Myeloid differentiation primary response 88 (MyD88) and TIR-domain-containing adapter-inducing interferon-β (TRIF) are two of the most well-studied adapter molecules that work in the TLR signaling pathway. In the MyD88-dependent pathway, the Interleukin-1 receptor-associated kinase (IRAK) proteins are phosphorylated, and they recruit and activate TNF receptor-associated factor-6 (TRAF6). TRAF6 coordinates the activation of mitogen-activated protein kinase (MAPK) and *nuclear factor kappa B* (NF-κB) pathways, which promote the production of proinflammatory cytokines and chemokines, and the activation of microbicidal mechanisms important for microbial elimination (43). The TRIF-dependent signaling leads to the activation of interferon regulatory factor (IRF) transcription factors responsible for the transcription of type I interferons and genes related to antiviral resistance, synthesis of proinflammatory cytokines, and regulation of immune responses (44). Given the importance of TLR signaling in host resistance against infections and inflammatory responses, it is crucial to understand the molecular mechanisms underlying the regulation of their intracellular signaling. Uncontrolled or excessive inflammation triggered by TLR may lead to tissue damage and increased susceptibility to infections, inflammatory disorders, and autoimmune diseases (45, 46).

TLR9 is a PRR that recognizes unmethylated cytidine-phosphate-guanosine (CpG) oligodeoxynucleotides derived from pathogens and responds to cellular components including proteins, nucleotides, and DNA, conferring protection against infections and maintaining homeostasis by removing cellular debris during physiological conditions. Uncontrolled or aberrant TLR9 signaling favors the development of inflammatory and autoimmune diseases during pathological cellular damage and stress signals (47). It was shown that Smurf1 is required for the negative regulation of TLR9-mediated inflammatory responses (**Figure 3**). During CpG-dependent TLR9 signaling, Smurf1 interacts with serine/threonine kinase 38 (Stk38), also known as NDR2, a kinase highly conserved from yeast to humans (48), and it facilitates Smurf1-mediated ubiquitination and degradation of mitogen-activated protein kinase kinase 2 (MEKK2). MEKK2 is a member of the MAP3K family of proteins that play important roles in TLR signal transduction (49). Smurf1 interaction with Stk38 was demonstrated to occur both in primary mouse peritoneal macrophages and in human embryonic kidney (HEK293) lineage cells. Smurf1-dependent ubiquitination and degradation of MEKK2 leads to reduced CpG-induced (but not LPS (*lipopolysaccharide*)-induced) activation of the extracellular signal−regulated protein kinase 1/2 (ERK1/2) MAPK and decreased production of tumoral necrosis factor (TNF) and IL-6 in macrophages. Mice deficient for Stk38 produce increased TNF and IL-6 levels and are more susceptible to *Escherichia coli* infection or sepsis induced by cecal ligation and puncture (CLP), compared to control littermates, due to uncontrolled systemic inflammation (50). In addition, it was shown that Smurf1 attenuates IL-17-induced IL-6, CXCL2, and CCL20 expression in both HeLa and HT-29 human lineage cells in a similar mechanism dependent on Smurf1 interaction with Stk38 and MEKK2 degradation (51). Taken together, these data suggest that Smurf1-dependent degradation of MEKK2 may be an important physiological mechanism to regulate inflammatory immune responses to avoid tissue damage.

*In vivo* ubiquitination assays demonstrated that Smurf1 also interacts with TRAF4 and TRAF6 to induce their ubiquitination and degradation in a ubiquitination-dependent manner (52, 53) (**Figure 3**). TRAF4 is described as a negative regulator of NF-κB signaling (54), and co-expression of Smurf1 with TRAF4 in HEK293 human cell line attenuates TRAF4’s negative effect on NF-κB activation (52). Similarly, HEK293 cells treated with IL-1β presented an attenuation in TRAF6 ubiquitination after Smurf1 knockdown, and an increased NF-κB activation (52). It suggests that Smurf1 may regulate innate immune signaling by targeting TRAF proteins for proteasome degradation.

Soluble mediators, including cytokines and eicosanoids, play a key role in regulating TLR signaling and inflammatory responses. Transforming growth factor beta (TGF-β) is a cytokine produced by many cell types in the body and it participates in key biological processes including cell growth, apoptosis, and proliferation. TGF-β is a critical anti-inflammatory factor that negatively regulates innate immune responses triggered by TLR agonists (55). It has been shown that Smurf1 is required for the anti-inflammatory effect of TGF-β through a mechanism dependent on the ubiquitination and degradation of the MyD88 adaptor (56) (**Figure 3**). Treatment of primary mouse peritoneal macrophages with TGF-β results in the degradation of endogenous MyD88, while the knockdown of Smurf1 abrogates TGF-β-dependent MyD88 degradation (56). Smurf1 interacts with MyD88 in CMT-93 mouse cell line under TGF-β-treatment and promotes MyD88 K48-linked ubiquitination and degradation (56). Interaction between Smurf1 and MyD88 is facilitated by Smad6, a protein that works in TGF-β-signaling (57), as the knockdown of Smad6 abrogated Smurf1-MyD88 interaction in mouse peritoneal macrophages. In addition, TGF-β loses its inhibitory activity when Smurf1 is knockdown in peritoneal macrophages treated with LPS (56). Thus, Smurf1 is required for the TGF-β-dependent negative regulation of inflammatory responses by targeting MyD88 for proteasomal degradation. It was recently shown that the negative regulation of MyD88 by Smurf1 may be exploited by pathogens to facilitate the establishment of infection. PPE36 is a 27-kDa cell-wall-associated protein expressed by *Mycobacterium tuberculosis*, the bacilli that cause human tuberculosis. PPE36 expression is enriched in *M. tuberculosis* virulent strains and is a potent inhibitor of NF-κB and MAPK pathways (58). It was shown that *M. tuberculosis* PPE36 facilitates the interaction between Smurf1 and MyD88 both in HEK293 human cell line and RAW264.7 mouse macrophage cell line overexpressing PPE36, resulting in increased K48-linked MyD88 ubiquitination and degradation in a Smurf1-dependent manner (58) (**Figure 3**). Accordingly, PPE36 depletion in *M. tuberculosis* leads to increased inflammation and decreased bacterial loads in the lungs of *M. tuberculosis*-infected mice (58), suggesting that PPE36-mediated Smurf1-dependent MyD88 degradation contributes to reduced inflammatory response and increased susceptibility to *M. tuberculosis* infection. Likewise, *M. tuberculosis* also secretes PtpA and Rv0222, two virulence factors capable to subvert the function and enzymatic activity of other host E3 ubiquitin ligases to favor its survival (59–61). These findings raise the possibility that besides *M. tuberculosis*, other pathogens may produce virulence factors that may subvert inflammatory immune responses through modulation of Smurf1 or other E3 ubiquitin ligases.

Innate immune responses triggered by TLR and other PRR lead to the secretion of TNF, a key proinflammatory cytokine with antimicrobial and antitumor activities (62, 63). It has been shown that Smurf1 may also regulate inflammatory responses by suppressing TNF transcription. Ubiquitin specific peptidase 5 (USP5), a deubiquitinase that is involved in multiple cellular processes such as DNA repair, reaction to stress, and cancer (64), is essential for the production of TNF (65). Smurf1 was shown to interact with USP5 and promote its proteasomal degradation in HEK293 human cell line (66) (**Figure 3**). As a consequence, Smurf1 repressed mRNA TNF transcription (66). Overall, Smurf1 works as a negative regulator of inflammatory innate immune responses by interacting with and stimulating proteasome-dependent degradation of molecules such as MyD88 and USP5.

IL-1β is an atypical proinflammatory cytokine secreted in response to the activation of inflammasomes, initially produced as an inactive cytosolic precursor (pro-IL-1β), which then suffers proteolytic cleavage to generate biologically active IL-1β (67). Pro-IL-1β processing is primarily mediated by the action of caspase-1, although other proteases derived from distinct cell sources have been reported as capable of cleaving pro-IL-1β (68). In absence of proper cell stimulation, the processing and release of IL-1β are inefficient, as intracellular unprocessed IL-1β products are degraded by the proteasome system (69). It was recently shown that Smurf1 participates in a delicate mechanism of regulation of IL-1β-dependent inflammation (70). Smurf1 interacts with intracellular pro-IL-1β in primary mouse macrophages treated with LPS and promotes both K63- and K48-linked ubiquitination of pro-IL-1β. While Smurf1-dependent K63-linked ubiquitination contributes to pro-IL-1β processing, K48-linked ubiquitination leads to pro-IL-1β proteasomal degradation (70), which suggests that Smurf1 may either stimulate or inhibit IL-1β secretion. It was further shown that Smurf1-pro-IL-1β interaction is enhanced by the interaction between Smurf1 and cyclophilin A (CypA), a peptidyl-prolyl isomerase that works on immune regulation (71, 72) (**Figure 3**). CypA-deficient mice treated with LPS present reduced lung injury at early stages of inflammation, followed by an increased lung injury at late stages of inflammation, in a mechanism dependent on the production of IL-1β (70). These data suggest that at early stages of inflammation, CypA promotes inflammation by increasing Smurf1-dependent pro-IL-1β processing, *via* K63-linked ubiquitination, while at late stages of inflammation, CypA inhibits inflammation by stimulating pro-IL-1β degradation by Smurf1 through K48-linked ubiquitination. Therefore, in addition to negatively regulating innate immune signaling, Smurf may promote inflammatory immune responses by stimulating CypA-dependent K63-linked ubiquitination of pro-IL-1β.

## 5 IFN-γ signaling

PRR signaling during innate immune responses promotes the release of cytokines required for cell recruitment and activation of adaptive immune responses. Interferon-gamma (IFN-γ) is a cytokine primarily secreted by natural killer (NK) cells and Th1 lymphocytes that has a key role to potentialize antimicrobial mechanisms mediated by innate immune cells. IFN-γ is a potent stimulator of antimicrobial mechanisms in macrophages including the production of reactive oxygen and nitrogen species, and antimicrobial autophagy (73–75). IFN-γ acts through a pathway dependent on the activation of the transcription factor signal transducer and activator of transcription 1 (STAT1) (76, 77). Smurf1 has been shown to interact with STAT1 to promote its K48-linked ubiquitination and proteasomal degradation in RAW264.7 mouse macrophage cell line treated with IFN-γ (40) (**Figure 3**). Smurf1 knockdown in peritoneal macrophages treated with IFN-γ increases the transcription of CXC chemokine ligand 9 (*Cxcl9*), CXC chemokine ligand 10 (*Cxcl10*), *Irf1*, and inducible nitric oxide synthase (*Nos2*) (40). Given the importance of IFN-γ in the regulation of innate immune responses and antimicrobial mechanisms, additional studies to better understand the interplay between Smurf1 and regulation of IFN-γ signaling using experimental models of infectious diseases might accelerate the development of host-directed therapies.

## 6 Antiviral immune responses

During viral infections, PRRs such as retinoic acid-inducible gene I (RIG-I), RIG-I-like receptors (RLR), and NOD-like receptors (NLR), recognize viral nucleic acid to trigger signaling cascades dependent on proteins including the adaptor protein mitochondrial antiviral signaling protein (MAVS) and the kinase TRAF-associated factor binding kinase 1 (TBK1). Activation of these proteins leads to the production of type I interferons and other cytokines, essential to drive/trigger antiviral responses (78). It has been shown that Smurf1 is required for the regulation of antiviral immune responses through the ubiquitination of members involved in the signaling cascades. Smurf1 interacts with MAVS, and overexpression of Smurf1 increased MAVS degradation in HEK293 human cell line in a mechanism dependent on the proteasome (79). The interaction between MAVS and Smurf1 is enhanced by NEDD4 family interacting protein 1 (Ndfip1), described as a recruiter and activator of members from the NEDD4 E3 ubiquitin ligases (80). Overexpression of Ndfip1 leads to increased Smurf1-dependent MAVS degradation and impairs the activation of several antiviral mechanisms both in mouse primary macrophages and HEK293 human cell line (79) (**Figure 3**). In addition to Ndfip1, it was recently reported that mammalian sterile 20-like kinase 4 (MST4), a ubiquitously expressed and highly conserved serine/threonine kinase of the MST family, enhances Smurf1-MAVS interaction, increases Smurf1-dependent MAVS degradation, and impairs type I IFN production in HEK293 cells transfected with polyino-sinic-polycytidylic acid (Poli (I:C)) or infected with Sendai Virus (81), in a similar mechanism as reported for Ndfip1 (**Figure 3**). Besides MAVS, Smurf1 interacts with STAT1 (40), a transcription factor required for antiviral immunity (82). Overexpression of Smurf1 in HEK293 cell line increases STAT1 degradation, and Smurf1-mediated STAT1 degradation impairs antiviral response in macrophages infected with vesicular stomatitis virus (VSV) (40) (**Figure 3**). Therefore, Smurf1 seems to work as a negative regulator of signaling pathways required for antiviral resistance. These findings raise the question of whether viruses could manipulate the inhibitory function of Smurf1 to favor their replication. Indeed, it was shown that infection with RNA viruses stimulates the expression of the enzyme OTU deubiquitinase1 (OTUD1), which interacts with Smurf1 and increases its intracellular expression in HEK293 lineage cells (83). In this condition, Smurf1 interacts not only with MAVS but with TRAF3 and TRAF6 as well, directing them to proteasomal degradation, which results in reduced antiviral response both in primary mouse macrophages and 2fTGH human lineage cells. Knockout mice for OTUD1 produce high levels of antiviral cytokines and are more resistant to infection with RNA viruses (83) (**Figure 3**). It suggests that an increased Smurf1-dependent inhibition of antiviral signaling is a mechanism that could be exploited by viruses to establish infection. New studies need to be conducted in order to evaluate whether RNA viruses could actively stimulate Smurf1 expression or manipulate other E3 ligase functions to favor their replication.

Deubiquitination enzymes work by removing ubiquitin chains from target substrates and are essential to fine-tune antiviral immune responses (84). Ubiquitin-specific protease 25 (USP25) is a deubiquitination enzyme expressed in most human tissues (85) that is capable to deubiquitinate TRAF3, TRAF6, and promoting host resistance to infection with DNA and RNA viruses (86). It was demonstrated that Smurf1 interacts with and induces USP25 K48-linked ubiquitination and degradation (**Figure 3**). In HEK293 cells infected with VSV, overexpression of Smurf1 results in increased viral replication, whereas USP25 overexpression leads to a decrease in viral replication (87). Thus, Smurf1 regulates antiviral immune responses by promoting ubiquitination and degradation of USP25.

Besides working on the regulation of antiviral immune responses, Smurf1 may target viral components to ubiquitin-dependent antiviral pathways. Smurf1 was identified as a factor required to mediate the selective delivery of virus components to the autophagy pathway (25). Smurf1 interacts with the Sindbis virus capsid protein in the cytoplasm of both mouse embryonic fibroblast and HeLa human lineage cells and promotes its delivery to autophagosomes for degradation (25) (**Figure 3**). In addition to components from the Sindbis virus, it was shown that Smurf1 interacts with the Spike protein from SARS-CoV-2 in HEK293 cells, and mRNA for Smurf1 was found to be overexpressed in swab samples from the nasopharynx and oropharynx of patients positive for COVID-19 (13) (**Figure 3**), which suggests that Smurf1 might present a relevant role to regulate immune resistance against COVID-19. To complete understand the role of Smurf1 in the regulation of antiviral host resistance, additional studies need to be carried out using experimental models of viral infections, or even drugs that target Smurf1 activity.

## 7 Antibacterial xenophagy

Autophagy is a cellular process in which components such as protein aggregates, misfolded proteins, and damaged organelles are sequestered in a double-layered vesicle, called autophagosome, for subsequent fusion with lysosomes for their degradation. Autophagy is a key mechanism to maintain cellular homeostasis, and its activation provides energy to cells when nutrients are scarce (88). It has been shown that autophagy may also target intracellular pathogens for lysosomal degradation, in a specialized type of autophagy known as xenophagy (89). One critical intracellular signaling that triggers xenophagy for the elimination of pathogens is the recognition of ubiquitin-bound pathogens by autophagy adaptors in the cytoplasm, followed by recruitment of the autophagy machinery, which commands the formation of the autophagosome membrane surrounding the microorganism. When bound to a ubiquitinated target, autophagy adaptors, including neighbor of BRCA1 gene 1 (NBR1), calcium-binding and coiled-coil domain-containing protein 2 (CALCOCO2, also known as NDP52), and sequestosome 1 (SQSTM1, also known as p62), couple to microtubule-associated protein light chain 3 II (LC3-II) (a protein present at the forming autophagosome membrane and commonly used as an autophagosomal marker (90)) to facilitate their delivering to autophagosomes for degradation. Therefore, the ubiquitination of intracellular pathogens is a crucial event required for the initiation of antimicrobial xenophagy (91, 92). It has been shown that Smurf1 is a host factor necessary to mediate ubiquitination and autophagic elimination of intracellular bacteria (26) (**Figure 3**). Mouse macrophages deficient for Smurf1 present defective ubiquitination of *M. tuberculosis*, reduced recruitment of the autophagy adaptor NBR1 to *M. tuberculosis*-containing structures, reduced targeting of *M. tuberculosis* to autophagosomes, and impaired capacity to contain *M. tuberculosis* replication (26). In addition to *M. tuberculosis*, mouse macrophages deficient for Smurf1 has a defective ability to control the replication of intracellular *Listeria monocytogenes* as well. Similarly, knockout mice for Smurf1 are more susceptible to *M. tuberculosis* infection with a high bacterial burden in the lungs and reduced survival in response to chronic mycobacterial infection. Interestingly, in addition to Smurf1 being highly expressed in lung biopsies of human patients infected with *M. tuberculosis* (26), it was shown that three Smurf1 polymorphisms were associated with a higher susceptibility to tuberculous meningitis (while they were not correlated to the severity or prognosis of tuberculous meningitis) in China (93), suggesting that Smurf1 might also participate in resistance against human tuberculosis. Despite the identification of Smurf1 as a factor required for mycobacterial xenophagy, the specific substrate(s) targeted by Smurf1 for ubiquitination remains to be discovered.

## 8 Concluding remarks and future perspectives

E3 ubiquitin ligases are a key family of proteins that functions in the post-transcriptional regulation of many cellular processes, including immune responses. Fine-tuning the intracellular pathways that work on inflammation, recognition of pathogens by innate immunity, or during physiological adaptation to pathological cell damage and stress is an essential condition to avoid the development of inflammatory diseases and autoimmunity. In this review, we highlighted the major identified functions of Smurf1 in the regulation of innate immune mechanisms related to the recognition of pathogens and elimination of microorganisms. With the exception of TRAF4, all Smurf1 substrates pointed in this review (MyD88, TRAF3, TRAF6, MEKK2, USP5, STAT1, MAVS) work on the positive regulation of innate immune signaling pathways and inflammation. Given that Smurf1 catalyzes the proteasomal degradation of innate immune-related substrates, future research focusing on identification of new Smurf1 substrates during innate immune responses and the search for pharmacological modulators of Smurf1’s activity could be a key strategy for the development of novel therapeutics against infectious, sterile inflammation, and autoimmune diseases. On one hand, to fight infectious diseases, strategies that aim at the positive regulation of innate immunity and inflammation may be successful. In such cases, the pharmacological inhibition of Smurf1 activity (94) might be able to stimulate inflammatory immune responses and drive the elimination of the pathogen. On the other hand, the search for new treatments for inflammatory and autoimmune diseases must be focused on the stimulation of intracellular synthesis of Smurf1 or on the positive regulation of its E3 ligase activity. Thus far, it has been identified a number of proteins capable of either regulating Smurf1 intracellular expression or Smurf1 ligase activity (95, 96). However, the effect of those proteins on the regulation of immune responses needs to be further investigated. In conclusion, additional studies using *in vivo* animal models of infectious and autoimmune diseases, in combination with pharmacological strategies, need to be conducted in order to define whether Smurf1could represent a therapeutic target for future clinical studies. Lastly, to better define the role of Smurf1 in human health and its potential as a therapeutic target, new genetic and functional studies with human samples need to be completed, as for now, most of the generated knowledge are based on human cell lines and mouse experimental models.

## Author contributions

All authors contributed to the manuscript preparation. LF developed the concept, general content, and structure of the figures. VC did a critical review of concepts regarding inflammation and antiviral immune responses. JA worked on the literature review and curation of articles used in manuscript preparation. LS-C and JA-C contributed equally to writing the manuscript. All the authors revised and approved the final version of the manuscript.
